# Brain Tumor Classification Using a Combination of Variational Autoencoders and Generative Adversarial Networks

**DOI:** 10.3390/biomedicines10020223

**Published:** 2022-01-21

**Authors:** Bilal Ahmad, Jun Sun, Qi You, Vasile Palade, Zhongjie Mao

**Affiliations:** 1 School of Artificial Intelligence and Computer Science, Jiangnan University, Wuxi 214122, China; bilalahmad@stu.jiangnan.edu.cn (B.A.); 7171905005@stu.jiangnan.edu.cn (Q.Y.); 7181905016@stu.jiangnan.edu.cn (Z.M.); 2 Centre for Computational Science and Mathematical Modelling, Coventry University, Coventry CV1 5FB, UK; ab5839@coventry.ac.uk

**Keywords:** variational autoencoder, generative adversarial networks, brain tumor classification, cancer classification, glioma, pituitary, meningioma, deep learning, convolutional neural networks, MRI, PET, radiolabeled PET

## Abstract

Brain tumors are a pernicious cancer with one of the lowest five-year survival rates. Neurologists often use magnetic resonance imaging (MRI) to diagnose the type of brain tumor. Automated computer-assisted tools can help them speed up the diagnosis process and reduce the burden on the health care systems. Recent advances in deep learning for medical imaging have shown remarkable results, especially in the automatic and instant diagnosis of various cancers. However, we need a large amount of data (images) to train the deep learning models in order to obtain good results. Large public datasets are rare in medicine. This paper proposes a framework based on unsupervised deep generative neural networks to solve this limitation. We combine two generative models in the proposed framework: variational autoencoders (VAEs) and generative adversarial networks (GANs). We swap the encoder–decoder network after initially training it on the training set of available MR images. The output of this swapped network is a noise vector that has information of the image manifold, and the cascaded generative adversarial network samples the input from this informative noise vector instead of random Gaussian noise. The proposed method helps the GAN to avoid mode collapse and generate realistic-looking brain tumor magnetic resonance images. These artificially generated images could solve the limitation of small medical datasets up to a reasonable extent and help the deep learning models perform acceptably. We used the ResNet50 as a classifier, and the artificially generated brain tumor images are used to augment the real and available images during the classifier training. We compared the classification results with several existing studies and state-of-the-art machine learning models. Our proposed methodology noticeably achieved better results. By using brain tumor images generated artificially by our proposed method, the classification average accuracy improved from 72.63% to 96.25%. For the most severe class of brain tumor, glioma, we achieved 0.769, 0.837, 0.833, and 0.80 values for recall, specificity, precision, and F1-score, respectively. The proposed generative model framework could be used to generate medical images in any domain, including PET (positron emission tomography) and MRI scans of various parts of the body, and the results show that it could be a useful clinical tool for medical experts.

## 1. Introduction

**Figure 1:**
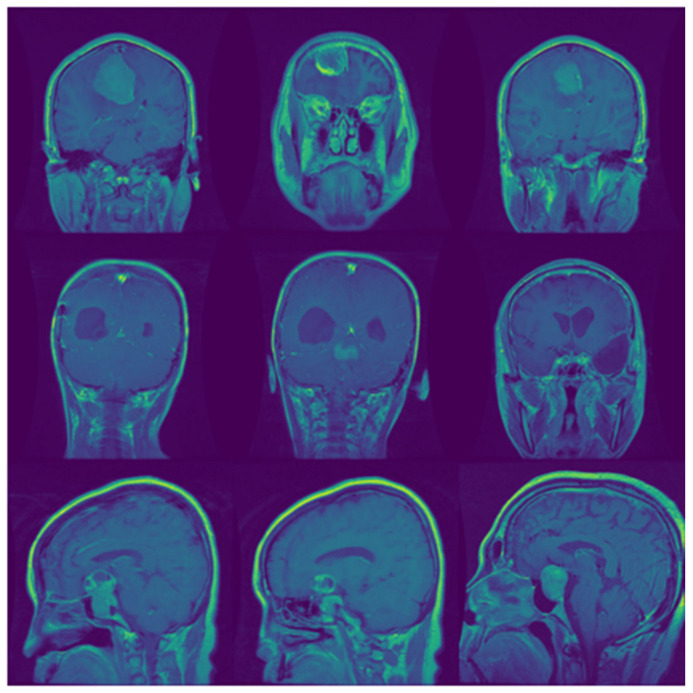
Sample images from the dataset. The first, second, and third rows of images represent glioma, meningioma, and pituitary brain tumors.

With all the wonderful progress of medicine over the last decades, some diseases are still life-threatening and, among them, brain cancer is the most aggressive [1]. Uncontrolled irregular growth of protein inside and around the brain tissues is known as a brain tumor. A brain tumor can be malignant or benign, malignant being the most aggressive type. In layman’s terms, the malignant type of brain tumor is called brain cancer. If a tumor breaches the covering and spreads into other parts, it is considered cancer [2]. Pituitary, meningioma, and glioma tumors are the three basic categories of brain tumors. The pituitary is a gland located at the base of the brain, and any abnormal growth of protein around this gland is known as a pituitary brain tumor [3]. Meningioma is a benign tumor that develops slowly and is found on the brain’s outer coverings beneath the skull [3]. The last and most aggressive one is glioma, with the highest mortality rate worldwide among all brain tumors [4]. It is commonly found in the cerebral hemispheres and the supporting tissue cells of the brain. Because of the location of the various brain tumors, pituitary and meningioma tumors are easy to detect, but gliomas are difficult to detect and analyze [3]. Sample images of glioma, meningioma, and pituitary from the dataset used in this research are presented in Figure 1.

Early symptoms of both benign and cancerous tumors are rare. The increased intracranial pressure is one of the initial symptoms. The skull bone restricts the amount of space available for growth. As a result, any new growth will raise intracranial pressure. Symptoms depend upon the site of the tumor; headache, vomiting, numbness of the hand or leg, or fits are a few symptoms [5].

Benign tumors, including meningioma and pituitary tumors, are slow-growing and typically cause no symptoms. However, neuropsychiatric symptoms such as anxiety, psychosis, personality changes, memory disturbances, or anorexia nervosa are common in patients with meningioma [6]. When only psychiatric symptoms are present, the diagnosis of meningioma could be delayed. Meningioma and almost all benign tumors are more likely to cause psychiatric symptoms and behavioral manifestations in individuals [7]. Gyawali et al. in [6] emphasize the need for neurological evaluation and neuroimaging in psychiatric patients, particularly those with unusual symptoms. Similarly, fatigue, seizures, edema, endocrinopathy, and psychiatric disorders are symptoms commonly found in patients with glioma tumors [8]. Because these symptoms are generic and not disease-specific, medical imaging is frequently used for brain tumor diagnosis.

Computed axial tomography (CT), positron emission tomography (PET), and magnetic resonance imaging (MRI) are a few common medical imaging techniques that are frequently used in medicine, including the diagnosis of brain tumors. In the clinical practice for the initial brain tumor diagnosis, computed axial tomography (CT) and magnetic resonance imaging (MRI) are the most widely used imaging techniques. Both CT and MRI have some advantages over each other. CT takes less time for imaging and offers high spatial resolution compared to MRI [9]. This property of CT makes it ideal for chest and bone-related diagnosis. However, the contrast of CT for soft tissue imaging is not high compared to MRI [9]. So, MRI is the most popular because of its high-resolution imaging capability.

In simple MRI scans, benign and malignant tumors look similar, and it is compulsory to differentiate among them at the initial stage of diagnosis. Contrast-enhanced MRI is the first choice of medical experts because of its ease of availability and better soft tissue resolution.

Although magnetic resonance imaging (MRI) has become the gold standard for diagnosing patients with tumors in any part of the body, classic MRI scans have two main limitations. It neither distinguishes neoplastic tissue from nonspecific, treatment-related changes after chemotherapy or surgery, nor does it show the tumor to the full extent [10]. Several modern MRI techniques, such as perfusion-weighted imaging and magnetic resonance spectroscopic imaging, are being tested recently in clinical practices to address the same diagnostic issues. Perfusion-weighted imaging highlights the fluids moving through the arteries, and diffusion-weighted imaging weights the MRI signal by the diffusion rate of water molecules [10].

Contrast-enhanced MRI plays a critical role in identifying, characterizing, and planning surgical tumor resection in patients with glioma. Any sign of contrast enhancement in early postoperative MRI (within 24–72 h) indicates incomplete resection [11]. A considerable number of patients could be misjudged with contrast-enhanced MRI, especially the patients with IDH-wildtype anaplastic glioma [12]. IDH (isocitrate dehydrogenase) is an important enzyme in the tricarboxylic acid cycle, and the tumors with normal IDH genes are referred to as “IDH wild-type” or “IDH negative” [13]. These IDH wild-type tumors are considered the most aggressive ones and lack contrast enhancement on MRI, so it may not be the best option for resection guidance [11]. Positron emission tomography (PET) scans have been considered recently in some clinical facilities to overcome the deficiency of contrast-enhanced MRI, particularly for the group of patients with IDH wild-type [11].

PET employs a range of radioactive tracers to target various metabolic and molecular processes. It can provide valuable extra information that enables medical experts to diagnose more precisely, particularly in ambiguous clinical scenarios [10]. For the diagnosis of most peripheral tumors in oncology, the most widely used PET tracer is 2-^18^F-fluorodeoxyglucose (^18^F-FDG) [10]. However, in the case of a brain tumor, the use of ^18^F-FDG PET is limited due to the high levels of glucose metabolism in normal brain tissues.

In cerebral gliomas, the proliferation marker ^18^F-3′-deoxy-3′-fluorothymidine (^18^F-FLT) accumulates in proportion to malignancy grade [14]. Nevertheless, ^18^F-FLT is unable to detect the full extent of a glioma because it cannot pass through the intact blood–brain barrier (BBB) and accumulates in portions of the tumor where the BBB has been disrupted.

The uptake of radiolabeled amino acids is poor in normal brain tissue, in contrast to the widely used ^18^F-FDG PET tracer, due to which tumors can be displayed with a strong tumor to background contrast. The ability of common amino acid tracers to penetrate through the intact BBB is one of their key characteristics, allowing for the depiction of the tumor that makes PET superior to contrast-enhanced MRI [11]. So, PET with radiolabeled amino acids is used as an alternative to the contrast-enhanced MRI for more exact tumor delineation [15]. The radiolabeled amino acid O-(2-[^18^F]fuoroethyl)-L-tyrosine (FET) is currently the most widely used tracer, particularly in Europe [10]. The fundamental advantage of PET employing radiolabeled amino acids is that their uptake is not affected by blood–brain barrier disruption, allowing it to detect tumor portions that are not visible on MRI [10,16].

Despite numerous technological advances in medical imaging and treatment, brain tumor patients’ survival rates remain extremely low [17]. No doubt, PET, radiolabeled PET, MRI, CT, and contrast-enhanced MRI help medical experts diagnose and classify brain tumors; however, accuracy is vulnerable to human subjectivity. Observing an enormous amount of medical data (MRI/CT images) is time-consuming for humans, and chances of human error are always there. The detection of a brain tumor at an early stage is crucial and depends upon the expertise of neurologists [18]. It is necessary to build computer-aided-diagnostic (CAD) systems that could help radiologists and other medical experts.

Researchers have shown great interest in developing automated AI-based intelligent systems. Traditional machine learning algorithms and methods for classifying brain tumors involve several steps, including heavy preprocessing, manual feature extraction, manual feature selection, classification, etc. Feature extraction and selection is a difficult process that requires prior domain knowledge, as the classification accuracy depends on good features being identified [19].

The problem of manual feature selection is eliminated with the arrival of deep learning. Image processing and deep learning methods have shown outstanding performance in various image-based tasks in various fields, including medicine [20,21,22,23].

Synthesized MRI/CT pictures can be extremely useful for training machine learning models when real MRI/CT images are prohibitively expensive to obtain from patients when considering time constraints and patient privacy [24].

Deep learning models feature hundreds of layers and millions of parameters. The more complex the model, the more data we need to train it. Overfitting is a prevalent problem when deep networks with a large number of parameters are trained on small datasets. The beauty of supervised deep learning lies in the quality and quantity of labeled data that is extremely difficult to acquire in the medical field.

In 2014, ground-breaking work in the field of generative models was proposed by Goodfellow et al., called generative adversarial networks (GANs) [25]. A GAN is made up of two components: a generator and a discriminator. The generator attempts to fool the discriminator by producing realistic-looking images, while the discriminator attempts to distinguish the created images as real or fake. They are alternately trained to reach final convergence. One significant difference between conventional generative models and GANs is that GAN learns the input distribution as a whole image instead of generating the image pixel by pixel.

So, researchers used GANs and tried to generate artificial medical images to overcome this problem. In the case of brain tumor magnetic resonance (MR) images, most GAN-based works are conducted to generate super-resolution brain MR images [26], some researchers used GANs for brain tumor segmentation [27,28], and very few used it for brain tumor classification [29].

In GANs and all its proposed extensions, there are a few things in common. First, all of them are the tools to generate those samples for which hundreds of thousands of images are available for training, e.g., MNIST. For medical image generation, we do not have that much training data generally.

Secondly, all these generative models use random Gaussian noise to sample the input vector. Because random Gaussian noise is a low-tailed distribution, the generator generates blurry and non-diverse images. Such image generation may not be helpful in the medical imaging field, as blurry images do not offer any realistic features to learn for the classifier.

In this paper, we tried to solve this problem by proposing a framework to generate brain tumor medical images artificially. This framework is the combination of two generative models, variational autoencoder (VAEs) and generative adversarial networks (GANs). We cascaded a GAN model with an encoder–decoder network trained separately on the training set and produced a noise vector with the image manifold information.

Our proposed method can generate realistic-looking sharp brain tumor images that improve the classification results significantly.

The rest of this paper is organized as follows. Section 1.1 reviews previous work related to brain tumor classification based on various machine learning methods, including GANs and its applications in medical imaging. Section 2 reports the proposed ED-GAN method in detail including experiment settings. Results & discussion, and conclusion are presented in Section 3 and Section 4, respectively.

### 1.1. Related Work

In the process of developing a machine learning-based intelligent system for the classification of brain tumors, researchers usually first perform segmentation of brain tumors by using various methods and then classify them [30]. This method improves the accuracy, but it is time consuming and takes one extra step before putting the network into the training. However, many researchers used CNNs to classify brain tumors directly without segmentation.

Justin et al. [31] used three classifiers (i.e., random forest (RF), a fully connected neural network (FCNN), and a CNN) to improve the classification accuracy. The CNN attained the highest rate of accuracy, i.e., 90.26%. Tahir et al. [30] investigated various preprocessing techniques in order to improve the classification results. They used three preprocessing techniques: noise reduction, contrast enhancement, and edge detection. The various combinations of these preprocessing techniques are tested on various test sets. They assert that employing a variety of such schemes is more advantageous than relying on any single preprocessing scheme. They used the Figshare dataset and tested the SVM classifier on it, which achieved 86% accuracy.

Ismael et al. [32] combined statistical features with neural networks. They extracted statistical features from the MR images for classification and used 2D discrete wavelet transforms (DWT) and Gabor filters for feature selection. They feed the segmented MR images to their proposed algorithm and obtain an average accuracy of 91.9%.

Another project that sought to categorize multi-grade brain tumors can be found in [33]. A previously trained CNN model is utilized along with segmented images to implement the method. They use three different datasets to validate the model. Data augmentation was performed using various techniques to handle the class imbalance and improve accuracy. Original and augmented datasets are tested on the proposed technique. In comparison to previous works, the presented results are convincing.

Nayoman et al. [34] investigated the use of CNNs and constructed seven different neural networks. One of the lightweight models performed best. Without any prior segmentation, this simple model achieves a test accuracy of 84.19%.

Guo et al. [35] propose an Alzheimer’s disease classifier. In Alzheimer’s disease, abnormal protein grows in and around the brain cells. The author uses graph convolutional neural networks (GCNNs) to classify Alzheimer’s disease into 2 and 3 categories. They used the Alzheimer’s Disease Neuroimaging Initiative (ADNI) dataset. The proposed graph nets achieved 93% for 2 class classification compared to 95% for ResNet architecture and 69% for SVM classifier. The proposed graph CNN achieved 77% in the three-class classification, ResNet 65%, and SVM 57%.

Ayadi et al. [36] used two different datasets, Figshare and Radiopaedia. One is used to classify brain tumor class, and the other is related to the classification of the stage of the brain tumor. For the classification of the main class of the tumor, they used a simple, lightweight CNN architecture.

Zhou et al. [37] used only axial slices from the dataset to classify the brain tumor. They also used a simple CNN classifier.

Pashaei et al. [38] proposed a method based on extreme learning machines in their study to classify the brain tumor. First, they extracted the features using CNN and used them in a kernel extreme learning machine (KELM) to build a classifier. KELM is famous for increasing the robustness of the classification task.

GAN-based networks for producing synthetic medical images have gained popularity in recent years due to their exceptional performance. A variation of Cycle GAN is proposed by Liu et al. [39] that generates Computed Tomography (CT) images using the domain control module (DCM) and Pseudo Cycle Consistent module (PCCM). The DCM adds additional domain information, while the PCCM maintains the consistency of created images. Shen et al. created mass images using GANs and then filled them with contextual information by incorporating the synthetic lesions into healthy mammograms. They asserted that their suggested network can learn real-world images’ shape, context, and distribution [40].

Chenjie et al. proposed a multi-stream CNN architecture for glioma tumor grading/subcategory grading that captures and integrates data from several sensors [41].

Navid et al. [29] proposed a new model for brain tumor classification using CNN on the Figshare dataset. They extracted the features by using the model as a discriminator of a GAN. Then a SoftMax classifier was added to the last fully connected layer to classify three tumors. They used data augmentation to improve the results and achieve 93.01% accuracy on the random split.

Other researchers have applied GANs to a variety of problems from medicine, including Shin et al. [42], who utilized a two-step GAN to generate MR images of brain parts with and without tumors [43], Ahmad used TED-GAN [44] to classify skin cancer images, and Nie [45] generated pelvic CT images.

GANs have gained the attention of researchers and are extensively used in a variety of medical imaging fields these days. Researchers attempt to improve results by utilizing complex and deep architectures. All these GAN-based studies contribute in various ways, but all of them used the random Gaussian noise as an input to the generator of the GAN. In the generative medical imaging field, manipulating the input noise of GANs is still un-explored.

## 2. Materials and Methods

### 2.1. Proposed Methodology

This section details our proposed framework. It combines two generative techniques, variational autoencoder and generative adversarial network, so we name it ED-GAN, where ED represents the encoder–decoder network. The variational autoencoder (VAE) consists of an encoder–decoder network. We first train a variational autoencoder (VAE) on our training set. After training the VAE, we swapped the encoder–decoder network into a decoder–encoder network. This swapping was inspired by [36]. Now the decoder will take the image as an input to generate the latent vector. The encoder will take this latent vector as an input and produce the noise. This noise is no longer random and has the information of the image manifold. After swapping, the decoder–encoder network follows the same process as the encoder–decoder networks but in the opposite direction.

In the next step, we used a generative adversarial network (GAN) that samples the noise vectors from the output of VAE (from the informative noise) instead of sampling from random Gaussian noise.

The proposed method, sampling the input noise from the trained decoder–encoder network, would avoid the GAN from mode collapse. Mode collapse is a common problem in GANs that occurs when GANs have limited training data, and GAN produces blurry and non-diverse images. The loss function keeps fluctuating with high variance in this situation. In our proposed method, there are negligible chances of mode collapse. Moreover, it readily adapts domain knowledge because its input is sampled from a latent vector of the trained VAE rather than random noise. Additionally, we added two conditional layers to ensure the proposed GAN produces images from all three classes of brain tumors.

**Figure 2:**
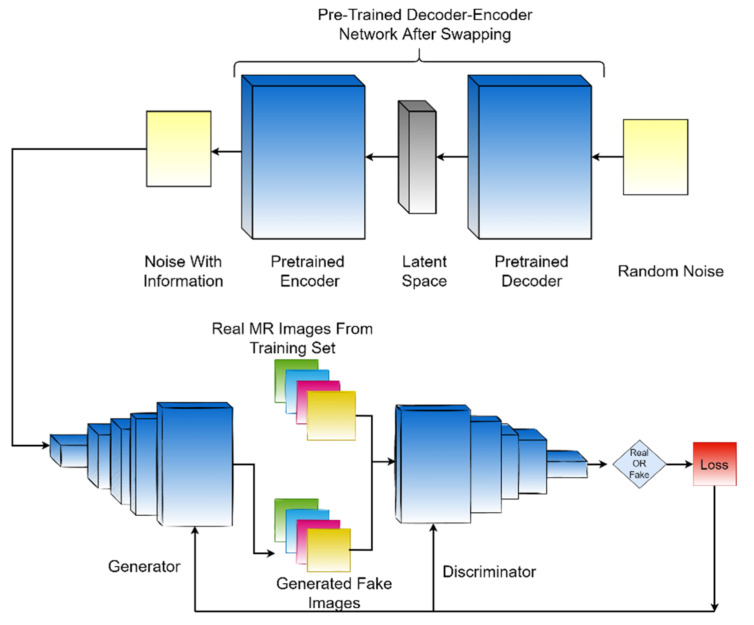
The main framework of the proposed methodology.

The whole framework is composed of a decoder–encoder network, one generative adversarial network, and a separate classifier (ResNet50) that uses the images generated by the proposed framework ED-GAN. for classification. The block diagram of the proposed framework is presented in Figure 2.

#### 2.1.1. VAE

The variational autoencoders (VAEs) consist of two parts, the encoder, and the decoder. The encoder consists of a separate network that takes the samples from the data ${\left\{{𝔁}_{i}\right\}}_{i=1}^{N}$ and tries to map it to the latent variables i.e., 𝓏. On the other hand, the decoder attempts to reproduce the input ${\left\{{\tilde{𝔁}}_{i}\right\}}_{i=1}^{N}$ with the help of learned distribution 𝓏. Input 𝓍 and reconstructed data samples $\tilde{𝓍}$ are in high dimensional space, whereas the latent variable 𝓏 is low dimensional comparatively. As the encoder and decoder are separate networks, their weights and biases are represented by ϑ  and  φ, respectively. Variational autoencoders have the same structure as deep autoencoders. However, variational autoencoders are based on the assumption that data (image) is generated by a directed model $P\left(𝓍|𝓏\right)$. Encoder learns the approximation $qᵩ\left(𝓏|𝓍\right)\;$ to the posterior distribution $P_{\vartheta }\left(𝓏|𝓍\right)$. In training variational autoencoders, we are interested in minimizing the loss function given in Equation (1) [46].

(1) $$L=-E_{𝓏\;∼\;qᵩ\left(𝓏|𝓍\right)}\left[\log P_{\vartheta }\left(𝓍|𝓏\right)\right]+D_{kl}\left[qᵩ\left(𝓏|𝓍\right)||P_{\vartheta }\left(𝓏\right)\;\right]$$

**Figure 3:**
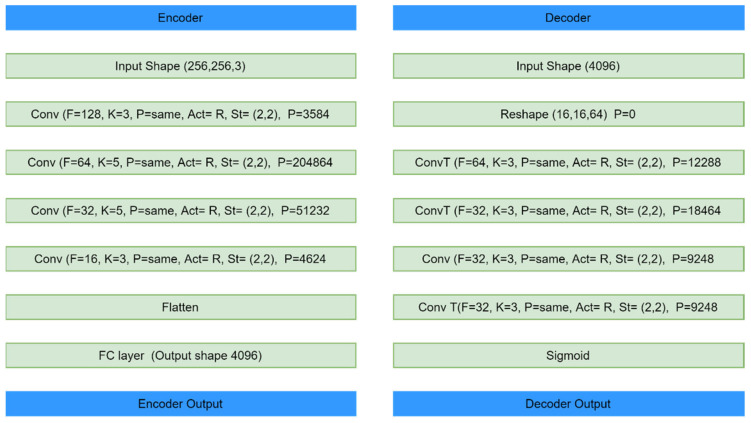
Variational autoencoder architecture used in this study.

Equation (1) represents the objective function of variational autoencoders, where the first term $E_{𝓏\;∼\;qᵩ\left(𝓏|𝓍\right)}\left[\log P_{\vartheta }\left(𝓍|𝓏\right)\right]$ represents the reconstruction likelihood, whereas the other term $D_{kl}\left[qᵩ\left(𝓏|𝓍\right)||P_{\vartheta }\left(𝓏\right)\right]$ ensures that a learned distribution q is similar to the prior distribution P. The architecture of VAE used for this study is shown in Figure 3.

#### 2.1.2. Encoder–Decoder Swapping

Let us say that $F_{1}$ and $F_{2}$ are the mapping functions of the encoder and decoder and ϑ and φ represents their mapping parameters, respectively. Then

$F_{1}\;:\;{𝔁}_{i}\to \;{𝔃}_{i}\;,\;{𝔁}_{i}∼P\left(𝔁\right),\;{𝔃}_{i}∼N\left(0,I\right),\;i=1,2,3,\ldots \;N$

$F_{2}:\;{𝔃}_{i}\to \;{\tilde{𝔁}}_{i}\;,\;{\tilde{𝔁}}_{i}∼P\left(𝔁|𝔃\right)$

From Equation (1)

(2) F1,F2= F1,F2argmin∑Dklqᵩ𝔃i|𝔁i||Pϑ𝔃i −E𝓏 ∼ qᵩ𝔃|𝔁logPϑ𝔁i|𝔃i

After swapping the encoder–decoder network into the decoder–encoder, we have:

$$F_{1}\;:\;{𝔃}_{i}\to {\tilde{𝔁}}_{i}\;,\;{𝔃}_{i}∼N\left(0,I\right),\;{\tilde{𝔁}}_{i}∼P\left(𝔁|𝔃\right)$$

$$F_{2}:\;{\tilde{𝔁}}_{i}\to \;{\tilde{𝔃}}_{i}\;,\;{\tilde{𝔃}}_{i}∼N\left(0,I\right)$$

where ${\tilde{𝔃}}_{i}$ is the noise distribution that contains the image manifold information, and the generative adversarial network would sample the input noise vector from this informative noise.

#### 2.1.3. Generative Adversarial Networks

Generative adversarial networks (GANs) have been one of the most impressive advancements in generative approaches. They are composed of two parts: a generative model (G) that approximates the data distribution and a discriminative model (D) that predicts whether the input sample came from the generative model or training data. Both discriminator and generator may be non-linear mapping functions, for example, a multi-layer perceptron. In our proposed method, the generator is forced to sample the noise vector from the noise generated by the pre-trained decoder–encoder network. It helps the generator to adopt the domain distribution quickly and avoid the mode collapse. The architecture of the generator and discriminator used for the generation of brain tumor images is shown in Table 1.

Generator G and discriminator D are trained concurrently: the parameters for generator G are adjusted to minimize $\log\left(1-D\left(G\left(𝓏\right)\right)\right)$ and the parameters for D are adjusted to minimize $\log\left(D\left(𝓍\right)\right)$, as though they were playing a two-player min–max game with a value function

$V\left(G,D\right)$:

(3) $$Min_{G}-Max_{D}\;V\left(G,D\right)=E_{𝓍\;∼\;P_{data}\left(𝓍\right)}\left[\log D\left(𝓍\right)]+E_{𝓏\;∼\;P_{𝓏}\left(𝓏\right)}\left[\log\left(1-D\left(G\left(𝓏\right)\right)\right)\right]\right]$$

#### 2.1.4. Adding Condition to the GAN

If both the generator G and discriminator D are provided with some additional information, such as class labels or input from other modalities, GANs can be expanded to a conditional model. Conditioning can be accomplished by feeding class labels into the discriminator and generator as additional input layers.

To ensure that the proposed network, ED-GAN, generates the images from a specific category, we provide additional information, the category label to the generator G and discriminator D. We denote the generator’s output as $G(𝓏|C_{lab})$, where “$C_{lab}“$ is the category label, auxiliary information provided to generator G and discriminator D as additional information. So, the loss function of the GAN with conditional information is presented in Equation (4).

(4) $$Min._{G}-Max._{D}\;V\left(G,D\right)=E_{𝓍\;∼\;P_{data}\left(𝓍\right)}\left[\log D(𝓍|C_{lab})]+E_{𝓏\;∼\;P_{𝓏}\left(𝓏\right)}\left[\log\left(1-D\left(G\left(𝓏|C_{lab}\right)\right)\right)\right]\right]$$

### 2.2. Experiment Settings

#### 2.2.1. Dataset

In this study, we used the public dataset proposed by Cheng [47]. It contains 3064 CE-MR images of three types of brain tumor (glioma, pituitary, and meningioma) from 233 patients. The images in this dataset are two-dimensional (2D-slices), not 3D volume images. This study included all three planes (axial, coronal, and sagittal) images from this dataset. A few sample images are depicted in Figure 1. Further details about the dataset and the training-test split are presented in Table 2.

#### 2.2.2. Hardware and Software

We used Ubuntu 18.04.2 LTS operating system supported by GeForce Nvidia GTX 1080 GPU (California, USA) and i7-6850 processor (California, USA). The code was written in PyCharm in python v3.8.0 with some external libraries, including Keras, TensorFlow v2.0, NumPy, Sci-Kit-learn, and Matplotlib.

#### 2.2.3. Performance Measures

We used precision, specificity, recall (sensitivity), average accuracy, and F1-score for performance measures. Among all, the most robust performance measure is F1-score in the classification tasks. Mathematically, sensitivity, specificity, precision, accuracy, and F1-score can be written as

(5) $$Sensitivity=\frac{TP}{TP+FN}\;$$

(6) $$Specificity=\frac{TN}{TN+FP}\;$$

(7) $$Precision=\frac{TP}{TP+FP}\;$$

(8) $$Accuracy=\frac{TP+TN}{Total\;samples}$$

(9) $$F1\;Score=2*\left(\frac{Sensitivity*Specificity}{Sensitivity+Specificity}\right)$$

where

TP = True Positive   FP= False Positive
FN = False Negative   TN = True Negative

Inception score [48] is one of the most widely used performance measures to evaluate the performance of GAN-based studies. It uses the Kullback-Leibler (KL) divergence that measures the difference between two probability distributions. We used some other generative models, GAN [25], DeliGAN [49], to compare the results. We use their public code with standard parameter values.

#### 2.2.4. Preprocessing and Hyperparameters

This section discusses preprocessing, including augmentation, pre-training of GAN, different optimizers, and learning rates.

Resizing the images, removing the duplicates, normalization, and augmenting the dataset are a few basic preprocessing steps that almost every machine learning engineer frequently performs in every task. Data augmentation is very crucial among them as it helps the deep learning models to prevent over-fitting. For instance, consider the AlexNet, the ImageNet Large Scale Visual Recognition Challenge (ILSVRC) winner in 2012; it could not achieve its reported maximum accuracy without augmentation for the ImageNet dataset. [50]. On the other hand, Paul et al. [31] obtained better results comparatively without preprocessing and augmentation. In one task, it played a very important role; on the other hand, in another task, augmentation was just another preprocessing step without any effect on the results. So, we took the augmentation as a hyperparameter and observed its effect on various experiment settings. We used only two kinds of augmentation in preprocessing, i.e., randomly rotating at different angles between 0–359^0^ and scaling the input images.

The ResNet50 architecture is used as the final classifier in this study. It has moderate depth, and due to its skip connection, it performs much better than the simple architecture of the same depth. We did not try other famous architectures for comparison as our main objective was to check whether the proposed method of GAN could improve the classification results. So, the training of GAN to produce better MR images of brain tumors was our main concern.

We trained ResNet50 on the images generated by the proposed generative network ED-GAN plus the training set (60% of the dataset). Before the final selection of hyperparameter values, we tested various values of several hyperparameters such as an optimizer, batch size, dropout rate and epoch, etc. Test results are discussed in the discussion section. Finally, we used Adam as an optimizer, with a learning rate of 0.0001, batch size 50, and with categorical cross-entropy.

## 3. Results and Discussion

In this study, we proposed the combination of two different generative models (VAEs and GANs) to generate artificial MR brain tumor MR images. Generating medical images using any generative model is time-consuming and more difficult compared to generating images of other species such as dogs, cats, and digits, where GANs are mostly used. Additionally, using these synthetic medical images to train the classifier for tumor identification is even more critical and requires a lot of strict evaluation before making any opinion about it. The dataset used in this study was somewhat small. We attempted to capitalize on the use of variational autoencoders in conjunction with GANs to handle the problem of the limited availability of data. We used the Figshare public dataset of brain tumor MR images [47]. The details of the dataset split are discussed in Section 2.2.1.

Before training the ResNet50 for a reasonable number of epochs, we trained it for 30 epochs under different values of hyperparameters, including different optimizers, batch size, and dropout rates. Table 3 summarizes the average accuracy for various optimizers under different optimizer learning rates. We chose the Adam optimizer to observe the effect of the dropout rate, as it performed better comparatively during the testing of the optimizer learning rate. Table 4 shows the effect of various dropout rates. To check the effect of the dropout rate, we fixed the epochs, learning rate, and optimizer to 30, Adam, and 0.0001, respectively. No generative images or augmentation was used for testing the hyperparameters; only the training set (60% of the dataset) was used. Augmentation of data plays a vital role in overcoming the class imbalance in the dataset and improving the results. We used plenty of generative images for augmentation; apart from this, we used the classic augmentation technique (rotation and scaling) to observe its effect on the results.

We observed an improvement of around 5% in the average accuracy when ResNet50 was trained on the training set with classic augmentation, without using generative images in the training set. Table 5 summarizes the results with and without augmentation.

In general, most past studies have relied solely on accuracy performance measures to compare the results with their proposed technique. However, using just accuracy for comparative purposes can be deceptive because it ignores other performance measures such as sensitivity, specificity, and precision. In the situation of imbalanced data, the accuracy of the classifier could be better for one class than the others. F1-score is a performance measure that includes all aspects (sensitivity and precision) of evaluations. This study used various performance metrics, including recall/sensitivity, specificity, precision, F1-score, and average accuracy.

**Figure 4:**
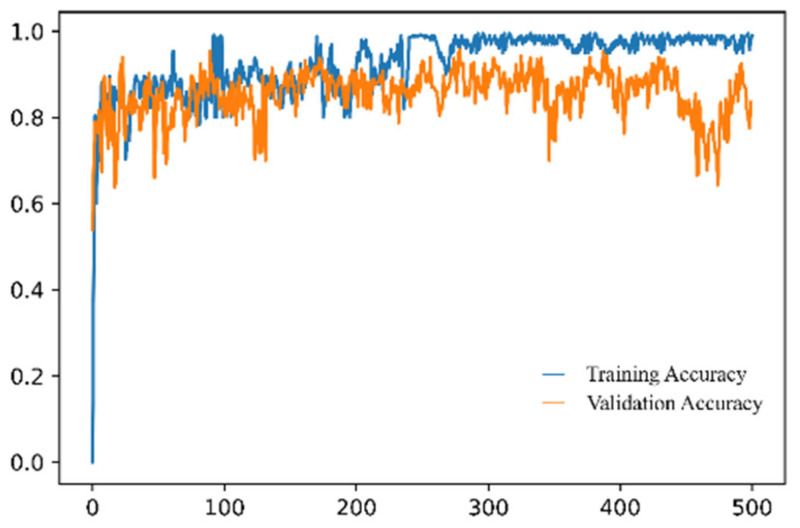
The training and validation accuracy of the classifier when the training set includes the images generated by ED-GAN.

Glioma is the most dangerous type of brain cancer. Neurologists are always interested in its sensitivity, specificity, and precision. ResNet50 trained on the Figshare dataset images only, without any generative images, achieved 82.86% sensitivity (recall) and 84.45% specificity for brain tumor class glioma. In contrast, the sensitivity and specificity improved to 96.50% and 97.52% for the same glioma class, respectively, when ResNet50 was trained with the images generated by the proposed method ED-GAN. All the hyperparameters values were the same, and the classifier was trained for 500 epochs. The training and validation accuracy graph of the classifier for 500 epochs is shown in Figure 4. A detailed quantitative comparison of sensitivity, specificity, precision, and F1-score for various experiments are summarized in Table 5.

**Figure 5:**
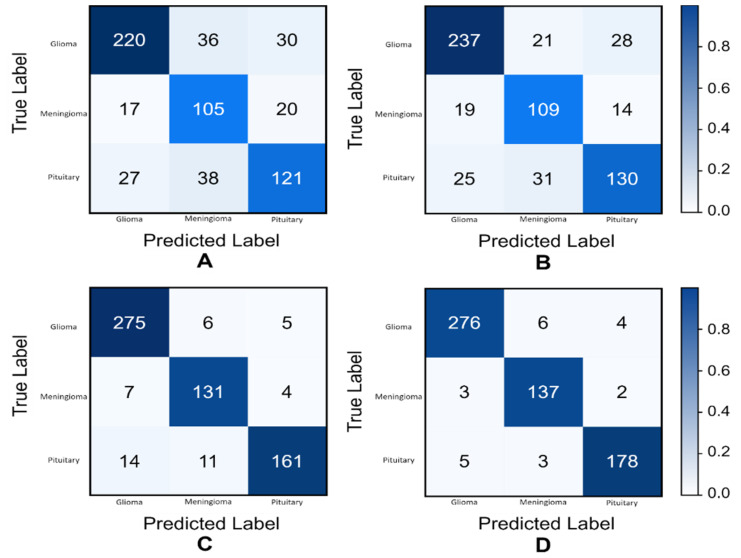
Confusion matrices of various experiments. (**A**): without classic augmentation and generative images, (**B**): with classic augmentation and without generative images, (**C**): without classic augmentation and with generative images, and (**D**): with generative images and with classic augmentation.

Figure 5 shows the confusion matrices of various experiments. A confusion matrix (CM) is a great way to see the behavior of the classifier visually. In a single glance, one can observe whether the classifier is biased to some dominant class or not.

Let us consider Figure 5A; the vertical and horizontal axis represents the true and predicted labels, respectively. If we consider a class glioma (test images of glioma = 286) and observe the matrix horizontally, the classifier predicted 220 images correctly as glioma. It incorrectly classified the remaining 66 images of glioma: 36 as meningioma and 30 as pituitary.

We used some other generative models, GAN [25], DeliGAN [49], to compare the performance of the proposed framework. The performance measure inception score [48] was used to measure the quality of generated images. We used the inception model to calculate the inception score, though it is not compulsory to use only this architecture. The inception score uses the KL divergence, which is a good performance measure for generative models. It measures the difference between two probability distributions instead of considering the image pixels only. To compare the classification results with these generative models, we used the ResNet50 as a classifier. ResNet50 is trained with the training set along with images generated by generative models GAN and DeLiGAN. We used generative images as augmentation, and did not use any other classic augmentation such as scaling, cropping or rotation, etc. Table 6 and Table 7 represent the comparison of inception score and classification performance measures for the proposed method with state-of-the-art generative models.

Apart from comparing with other image generative methods, we compare our classification results with several other studies published in various journals within the last five years. We selected 11 studies for comparison. They all used the same public dataset of brain tumor MR images. Out of the 11 studies, 9 reported an average accuracy of more than 90%. The average accuracy of the proposed framework is better, around 2–7%. The comparative classification results and other insightful information are summarized in Table 8.

**Figure 6:**
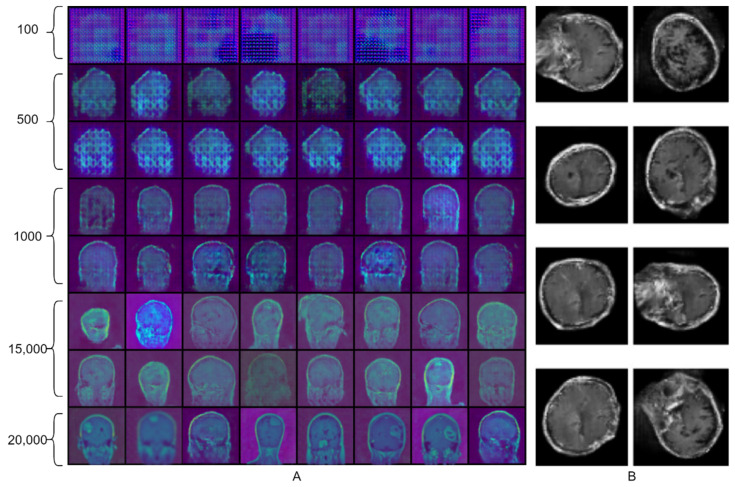
Artificially generated brain tumor MR images. (**A**) MR images of brain tumor generated by proposed ED-GAN. The top and the bottom rows represent the images generated by the proposed method after 100 and 20,000 training steps, respectively. (**B**) Brain tumor images generated by Ghassemi et al. [29]. They used the same public dataset as ours for the training of their proposed generative model. They reported an average accuracy of around 95%, only 2% less than we achieved in this study. However, there is much quality difference in the generated images of both methods.

GAN-based generative models can easily learn the outer features, such as the shape of the skull, but it is quite challenging to generate fine features by using GAN, such as tumors inside the skull. We can observe this situation in Figure 6B. This Figure is taken from [29], where they used the GAN for the pre-training of brain tumor classifier and achieved an average accuracy of around 95%. Figure 6A represents the images generated by the proposed ED-GAN. Here, we can clearly observe the quality difference of generated images of brain tumors. Our proposed extension of GAN, ED-GAN, could generate better images because it samples the noise from the informative noise vector instead of random Gaussian noise. Furthermore, this is the quality of generated images that ensured the proposed framework achieved a better average accuracy of 96.25% on the test set.

## 4. Conclusions

This paper proposed a framework that is the combination of two distinct generative models, an encoder–decoder network and a generative adversarial network. We trained the encoder–decoder network separately and swapped it to a decoder–encoder network. The output of this swapped network is a noise, not a reconstructed image. This output noise has the information of the domain, and we let the generative adversarial network sample the input noise vector from this informative noise instead of random Gaussian noise. Because of the use of the information noise in the GAN, there were very small chances of mode collapse, and it generated realistic-looking brain tumor images from all three classes. We used these generated images and the original training set in the ResNet50 classifier training.

The use of generated images by our proposed method ED-GAN improved the average accuracy from 72.63% to 96.25%. Other performance measures, sensitivity (recall), specificity, precision, and F1-score, also improved. Moreover, we compared the results with several existing studies related to brain tumor classification. Results proved that the proposed framework could be used as a clinical tool for neurologists and various other medical experts, as the proposed method can be used to generate medical images in other domains, not only for brain tumors.

## Figures and Tables

**Table 1:** Generator and discriminator architectures used in this research.

Generator			Discriminator		
Layer type	Output Shape	Par.	Layer type	Output Shape	Par.
Dense	1310720	132382720	Input 256x256		
LReLU	1310720	0	Con2D	128, 128, 128	1280
Reshape	32, 32, 1280	0	LReLU	128, 128, 128	
Con2DT	64, 64, 1280	26215680	Con2D	64, 64, 64	73792
LReLU	64, 64, 1280	0	LReLU	64, 64, 64	
Con2DT	128, 128, 1280	26215680	Con2D	32, 32, 32	18464
LReLU	128, 128, 1280	0	LReLU	32, 32, 32	0
Con2DT	256, 256, 1280	26215680	Dropout	32, 32, 32	0
LReLU	256, 256, 1280	0	Flatten	32768	
Con2d	256, 256, 1	1310721	Dense	None, 1	32769
Total params: 212,340,481			Total params: 126,305		
Trainable params: 212,340,481			Trainable params: 126,305		

LreLu = LeakyReLU   Par. = Parameters  Con2DTr = Conv2DTranspose.

**Table 2:** Dataset division into training, validation, and test sets.

Tumor Type	Number of Slices			Tran. Set	Val. Set	Test Set
	View	Slices		60%	20%	20%
			Total			
Glioma	Sagittal	495	1426	855	285	286
	Coronal	437				
	Axial	494				
Meningioma	Sagittal	231	708	424	142	142
	Coronal	268				
	Axial	209				
Pituitary	Sagittal	320	930	558	186	186
	Coronal	319				
	Axial	291				

Tran. = Training Val. = Validation.

**Table 3:** Effect of various learning rates on different optimizers.

Various ML Optimizers and Their LR						
	1	0.5	0.1	0.01	0.001	0.0001
SGD	30.45	43.2	62.49	65.25	69.7	70.15
Adagrad	45.12	51.46	65.23	65.5	69.15	68.75
RMSprop	55.27	56.7	60.61	61.45	65.28	63.32
Adam	53.5	60.3	65.8	66.1	69.36	72.45

ML = Machine Learning, LR = Learning Rates.

**Table 4:** Effect of dropout rate on average accuracy.

Droupout Rate	0.1	0.25	0.5	0.75	0.9
Accuracy %	71.10	71.52	69.75	45.78	30.15

**Table 5:** The classification results of brain tumors under various experiment settings.

Without Classic Augmentation and Generative Images				
	**Glioma**	**Meningioma**	**Pituitary**	**Average. Accuracy**
Recall	0.7692308	0.7394366	0.6505376	72.63%
Specificity	0.837037	0.8216867	0.8666667	
Precision	0.8333333	0.5865922	0.7076023	
F1-Score	0.8001	0.6542056	0.6778711	
**With Classic Augmentation and Without Generative Images**				
Recall	0.828671	0.767606	0.698925	77.52
Specificity	0.844523	0.875895	0.891753	
Precision	0.843416	0.677019	0.755814	
F1-Score	0.835979	0.719472	0.726257	
**Without Classic Augmentation and With Generative Images**				
Recall	0.961538	0.922535	0.865591	92.3
Specificity	0.932907	0.962472	0.978313	
Precision	0.929054	0.885135	0.947059	
F1-Score	0.945017	0.903448	0.904494	
**With Generative Images and With Classic Augmentation**				
Recall	0.965035	0.964789	0.956989	96.25
Specificity	0.975232	0.980562	0.98568	
Precision	0.971831	0.938356	0.967391	
F1-Score	0.968421	0.951389	0.962162	

Classic augmentation includes rotation and scaling. “With Generative Images” means images artificially generated from the proposed framework ED-GAN are included in the training set of the classifier (ResNet50).

**Table 6:** Inception score obtained by DeLiGAN, GAN, and ED-GAN.

Generative Models	Inception Score
GAN [25]	1.845 ± 0.084
DeLiGAN [49]	2.102 ± 0.091
ED-GAN (proposed)	**2.457 ± 0.012**

**Table 7:** The comparison of classification results of the proposed model with GAN and DeLiGAN.

	Glioma	Meningioma	Pituitary	Average Accuracy
**GAN** [25]				
Recall	0.839160	0.866197	0.801075	83.3%
Specificity	0.894736	0.919621	0.909774	
Precision	0.882352	0.783439	0.805405	
F1-Score	0.860215	0.822742	0.803234	
**DeLiGAN** [49]				
Recall	0.870629	0.908450	0.811827	86.15%
Specificity	0.927152	0.915331	0.935643	
Precision	0.918819	0.777108	0.853107	
F1-Score	0.894075	0.837662	0.831955	
**ED-GAN (Proposed)**				
Recall	0.961538	0.922535	0.865591	92.3%
Specificity	0.932907	0.962472	0.978313	
Precision	0.929054	0.885135	0.947059	
F1-Score	0.945017	0.903448	0.904494	

**Table 8:** Comparison of the proposed methodology results with several existing studies that used the same dataset.

Method	Year	No of Images	Manual Segmentation	Best Average. Accuracy
Bayesian Approach [51]	2020	3064	Yes	73.9
Capsule Network [52]	2019	3064	Boundary Box	90.89
Features Extraction [47]	2015	3064	Yes	91.28
CNN [31]	2017	3064	Yes	72.13%
CNN [36]	2020	3064	No	94.7%
SVM [30]	2019	3064	No	86.0%
DWT-GABOR-NN [32]	2018	3064	Yes	91.9%
CNN [37]	2018	989	No	92.13%
Ensemble CNN [38]	2018	3064	Not mentioned	93.68
GAN-Based (Random Split) [29]	2019	3064	No	95.6
GAN-Based [29]	2019	3064	No	93.01
ED-GAN (Proposed)	2021	3064	No	Without Aug. = 92.3%’ With Aug. = 96.25%

## Data Availability

We used the pubic dataset from https://journals.plos.org/plosone/article?id=10.1371/journal.pone.0144479 accessed on 5 September 2021.
